# Activation of RAR*α* induces autophagy in SKBR3 breast cancer cells and depletion of key autophagy genes enhances ATRA toxicity

**DOI:** 10.1038/cddis.2015.236

**Published:** 2015-08-27

**Authors:** D Brigger, A M Schläfli, E Garattini, M P Tschan

**Affiliations:** 1 Division of Experimental Pathology, Institute of Pathology, University of Bern, Bern, Switzerland; 2 Graduate School for Cellular and Biomedical Sciences, University of Bern, Bern, Switzerland; 3 Laboratory of Molecular Biology, IRCCS-Istituto di Ricerche Farmacologiche ‘Mario Negri', Milano, Italy

## Abstract

All-*trans* retinoic acid (ATRA), a pan-retinoic acid receptor (RAR) agonist, is, along with other retinoids, a promising therapeutic agent for the treatment of a variety of solid tumors. On the one hand, preclinical studies have shown promising anticancer effects of ATRA in breast cancer; on the other hand, resistances occurred. Autophagy is a cellular recycling process that allows the degradation of bulk cellular contents. Tumor cells may take advantage of autophagy to cope with stress caused by anticancer drugs. We therefore wondered if autophagy is activated by ATRA in mammary tumor cells and if modulation of autophagy might be a potential novel treatment strategy. Indeed, ATRA induces autophagic flux in ATRA-sensitive but not in ATRA-resistant human breast cancer cells. Moreover, using different RAR agonists as well as RAR*α*-knockdown breast cancer cells, we demonstrate that autophagy is dependent on RAR*α* activation. Interestingly, inhibition of autophagy in breast cancer cells by either genetic or pharmacological approaches resulted in significantly increased apoptosis under ATRA treatment and attenuated epithelial differentiation. In summary, our findings demonstrate that ATRA-induced autophagy is mediated by RAR*α* in breast cancer cells. Furthermore, inhibition of autophagy results in enhanced apoptosis. This points to a potential novel treatment strategy for a selected group of breast cancer patients where ATRA and autophagy inhibitors are applied simultaneously.

Macroautophagy (hereafter referred to as autophagy) is a conserved mechanism characterized by the formation of double-membrane structures. These so-called autophagosomes deliver cytoplasmic material to the lysosome for subsequent degradation.^1^ Basal autophagy requires tight regulation as alterations in autophagy have been associated with many pathological conditions, including cancer.^2^ In addition, autophagy has been linked to fundamental processes such as development and cellular differentiation. In these processes, autophagy contributes to cell remodeling as observed during erythrocyte, lymphocyte or adipocyte differentiation.^3^ In the context of cancer and cancer therapy, autophagy is a double-edged sword. Owing to its homeostatic role in the removal of potentially harmful damaged organelles and protein aggregates, it is suggested to be tumor suppressive under normal conditions.^4^ In cancer cells, however, autophagy can be oncogenic, enabling survival under stressful conditions.^5^ Hence, the role of autophagy in tumorigenesis is clearly dependent on the cellular context and the tumor stage. In some cases, therapeutic agents induce an autophagic response that can promote resistance to treatment. In other cases, autophagy contributes to the action of antitumor agents.^6^ Therefore, knowledge about the action exerted by autophagy in response to anticancer treatments is a prerequisite for the identification of patients benefiting from therapeutic strategies based on autophagy modulators.

All-*trans* retinoic acid (ATRA), the active metabolite of vitamin A, exerts diverse functions in almost every cell and organ system. ATRA controls cell proliferation, differentiation as well as immune, and neuronal functions primarily via regulation of gene expression.^7^ Endogenous retinoid levels are altered in different diseases of the lung, kidney and central nervous system, and contribute to their pathophysiology.^8^ ATRA is successfully used in the treatment of acute promyelocytic leukemia (APL), where it induces granulocytic differentiation of the blast and subsequent cell death of the differentiated leukemic cells. Importantly, ATRA-induced differentiation of the APL cell line, NB4, involves induction of macroautophagy.^9, 10, 11, 12^ In addition to its cytodifferentiating capacity in APL, ATRA has been proposed as an antitumorigenic agent for other types of cancer. The antiproliferative, cytodifferentiating and proapoptotic effects of retinoids are predominantly mediated by the nuclear hormone retinoid acid receptors RAR*α*, RAR*β* and RAR*γ.*^13, 14^ In breast cancer, preclinical studies have shown that retinoids are promising therapeutic agents. However, the clinical trials conducted so far were somewhat disappointing, possibly as a consequence of the study designs.^15^ Breast cancer is a highly heterogeneous disease represented as a collection of diseases with distinct histopathological and molecular features. The most important clinical classification of this tumor is based on the determination of ER (estrogen receptor), PR (progesterone receptor) and HER2 (human epidermal growth factor receptor-2) receptors. ER-positive breast cancer patients are eligible for hormonal therapies, whereas HER2 oncogenic activity can be blocked using targeted therapies.^16^ Approximately 15–20% of breast carcinomas overexpress HER2, which is associated with poor prognosis.^17^ Owing to the development of resistance to current HER2-targeted treatments such as trastuzumab and lapatinib alternative therapeutic strategies are required.^18, 19^ ATRA was recently shown to exert strong antitumor activity in cell lines representing a subgroup of HER2-positive breast tumors characterized by coamplification of the *ERBB2* and *RARα* genes.^20^ This antitumor activity is remarkably stimulated by simultaneous HER2 inhibition with lapatinib. In addition, autophagy is induced upon ATRA treatment of the APL-derived cell line NB4^9, 10, 11^ and retinoids have clinical relevance in breast cancer. Thus, we investigated whether and how autophagy is induced in breast cancer cells. In addition, we evaluated whether autophagy modulation represents a potential therapeutic strategy for potentiating ATRA cytotoxicity in breast cancer cells.

## Results

### ATRA initiates a dose- and time-dependent autophagic response associated with epithelial differentiation in SKBR3 cells

**Figure 1:**
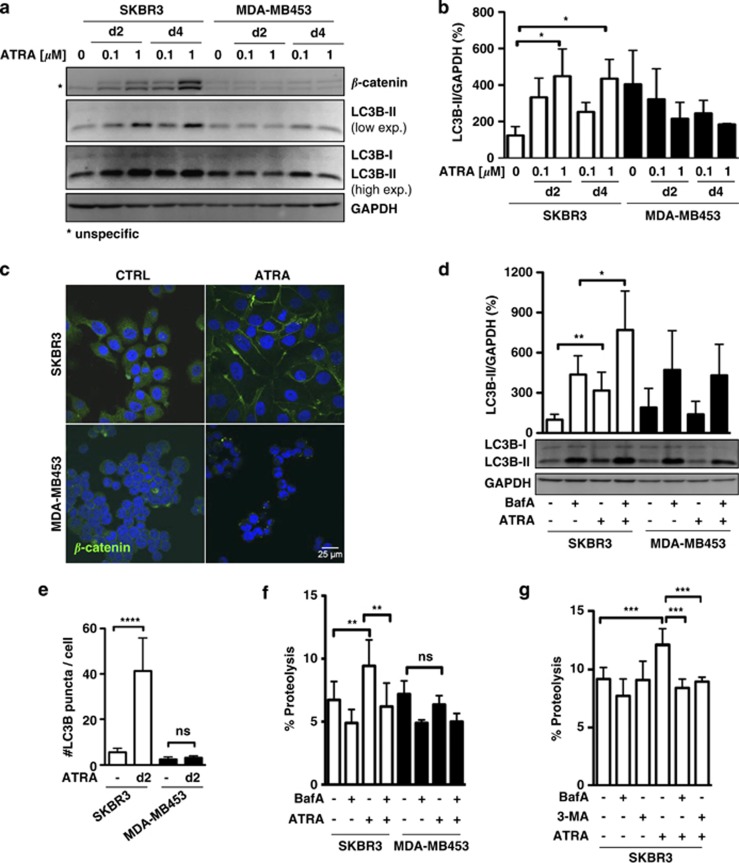
ATRA induces autophagy in SKBR3 cells. (**a**) SKBR3 and MDA-MB-453 breast cancer cells were treated either with 0.1 or 1 *μ*M ATRA for 2 and 4 days, respectively. *β*-Catenin and LC3B levels were measured by western blotting. GAPDH was used as a loading control. (**b**) LC3B-II quantification from at least three independent experiments. LC3B-II expression was normalized to GAPDH and to vehicle-treated SKBR3 cells. (**c**) *β*-Catenin staining of SKBR3 and MDA-MB453 cells. Cells were control or ATRA (1 *μ*M) treated for 2 days. *β*-Catenin FITC (fluorescein isothiocyanate) and nuclear DAPI (4',6-diamidino-2-phenylindole) staining as analyzed by confocal microscopy are shown. (**d**) Quantification of endogenous LC3B puncta. LC3B in SKBR3 and MDA-MB453 cells treated with 1 *μ*M ATRA for 2 days. (**e**) LC3B-II western blotting and quantification of the autophagic activation upon treatment with 1 *μ*M ATRA for 2 days in the presence or absence of BafA for 2 h at 200 nM. LC3B-II levels were normalized to GAPDH and to vehicle-treated SKBR3 cells. Standard deviations for five independent experiments are shown. (**f**) Long-lived protein degradation assay of SKBR3 and MDA-MB453 cells treated as in (**e**). Radioactivity was determined by liquid scintillation counting of at least three independent experiments. Absolute proteolysis is shown. (**g**) Long-lived protein degradation assay of SKBR3 treated as in (**e**) and including treatment with the macroautophagy-specific inhibitor 3-MA (10 mM). Analysis as in (**f**). Mann–Whitney *U-*test: *P<0.05, **P<0.01, ***P<0.001 and ****P<0.0001

To determine whether ATRA modulates autophagy in breast cancer cells, we first measured steady-state levels of the autophagy marker LC3B-II in the two luminal, HER2-positive ER-negative breast cancer cell lines, SKBR3 (HER2/RAR*α* coamplification) and MDA-MB453 (HER2 amplification), upon challenge with different concentrations of ATRA during different time periods. We chose these two cell lines as SKBR3 are sensitive to and MDA-MB453 cells are resistant to ATRA.^20^ In the SKBR3 cells, we observed a dose-dependent increase in the steady-state levels of lipidated LC3B (LC3B-II), which was paralleled by induction of the differentiation-associated *β*-catenin protein. These effects were not observed in ATRA-treated MDA-MB453 cells (Figures 1a and b). ATRA-dependent cytodifferentiation was further visualized at day 2, using immunofluorescence microscopy to highlight the redistribution of *β*-catenin to the cell membrane where it contributes to epithelial differentiation.^20^ ATRA treatment of SKBR3 clearly caused relocalization of *β*-catenin to the cell membrane. Regardless of ATRA treatment, MDA-MB453 cells showed a punctuated perinuclear distribution with no membrane localization of *β*-catenin^20^ (Figure 1c).

**Figure 2:**
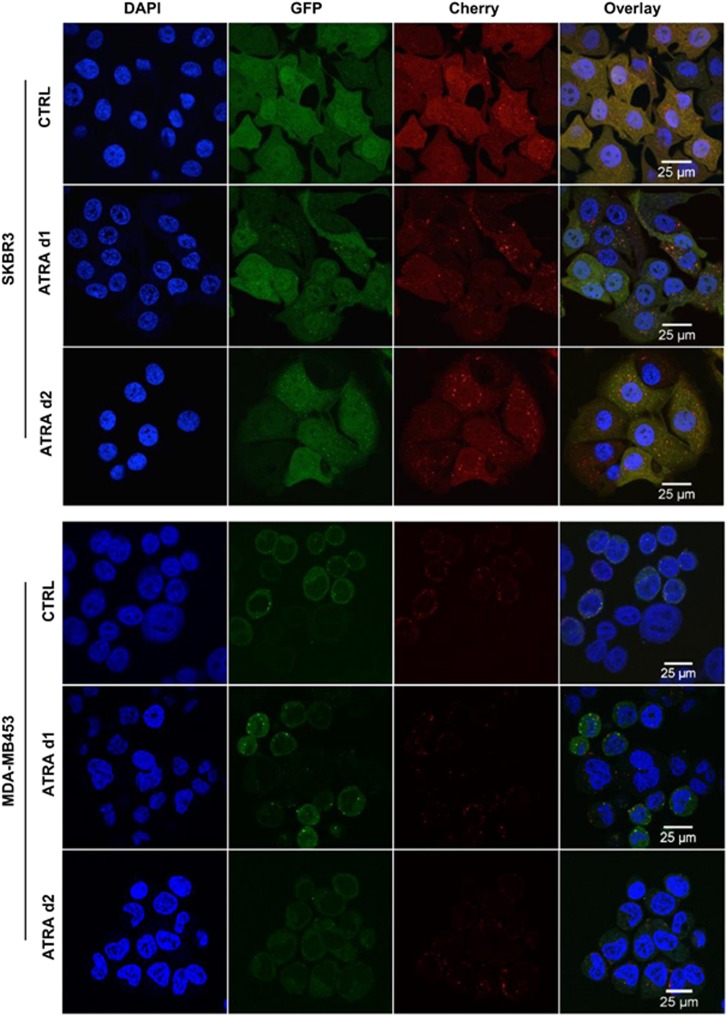
Autophagic flux in ATRA-treated SKBR3 cells. Stable Cherry-GFP-LC3B-expressing SKBR3 and MDA-MB453 cells were treated with 1 *μ*M ATRA for 1 and 2 days. Changes in autolysosome formation is shown by confocal microscopy analysis. Green (GFP) and red fluorescence (Cherry) signals, as well as an overlay thereof are shown. Representative pictures from three independently performed experiments are shown

To further quantify autophagy induction in our cell line models, we determined endogenous LC3B puncta by confocal microscopy. We observed a significant increase in endogenous LC3B puncta in SKBR3 but not in MDA-MB453 cells (Figure 1d and Supplementary figure 1a). Given the significant induction of LC3B-II (Figures 1a and b), we next monitored autophagic flux to exclude that a block in the autophagy pathway causes LC3B-II accumulation. We therefore quantified lipidated LC3B levels in the presence and absence of a saturating lysosomal inhibitor, bafilomycin A1 (BafA). ATRA-induced LC3B lipidation, which was significantly enhanced in the presence of BafA, suggests increased autophagic flux. In contrast, ATRA administration did not result in a further increase beyond basal autophagy levels in the MDA-MB453 cell line (Figure 1e). As autophagy must be evaluated using different techniques,^21^ we also used a standard biochemical method to determine autophagic activity. We measured the turnover of long-lived ^14^C-valine-labeled proteins upon 2 days of ATRA treatment of SKBR3 and MDA-MB453 cells in the presence or absence of the autophagy inhibitor BafA. In SKBR3 cells, the proteolysis of long-lived proteins was significantly increased by ATRA, whereas no such increase was observed in MDA-MB453 cells (Figure 1f). Moreover, 3-methyladenine (3-MA) blocked ATRA-mediated autophagy as efficiently as BafA, indicating that retinoids preferentially induce macroautophagy in retinoid-sensitive breast cancer cells (Figure 1g). As a third assay to assess autophagic flux in our breast cancer cells, we took advantage of a Cherry-GFP-LC3B tandem construct. As the GFP signal is quenched at low pH, this system allows to distinguish between autophagosomes (Cherry^+^/GFP^+^) and autolysosomes (Cherry^+^).^22^ ATRA treatment caused an increase of Cherry^+^/GFP^+^ dots as well as Cherry^+^ dots in SKBR3 cells clearly indicating activation of autophagic flux (Figure 2, upper panel). In contrast, ATRA treatment caused a decrease in the number of autophagosomes (Cherry^+^/GFP^+^) and autolysosmes (Cherry^+^) over time in MDA-MB453 cells. Altogether, these different standard assays to monitor autophagy convincingly demonstrate that ATRA activates autophagic flux in ATRA-sensitive SKBR3 but not in ATRA-resistant MDA-MB453 cells.

### ATRA-induced autophagy flux depends on RAR*α*

**Figure 3:**
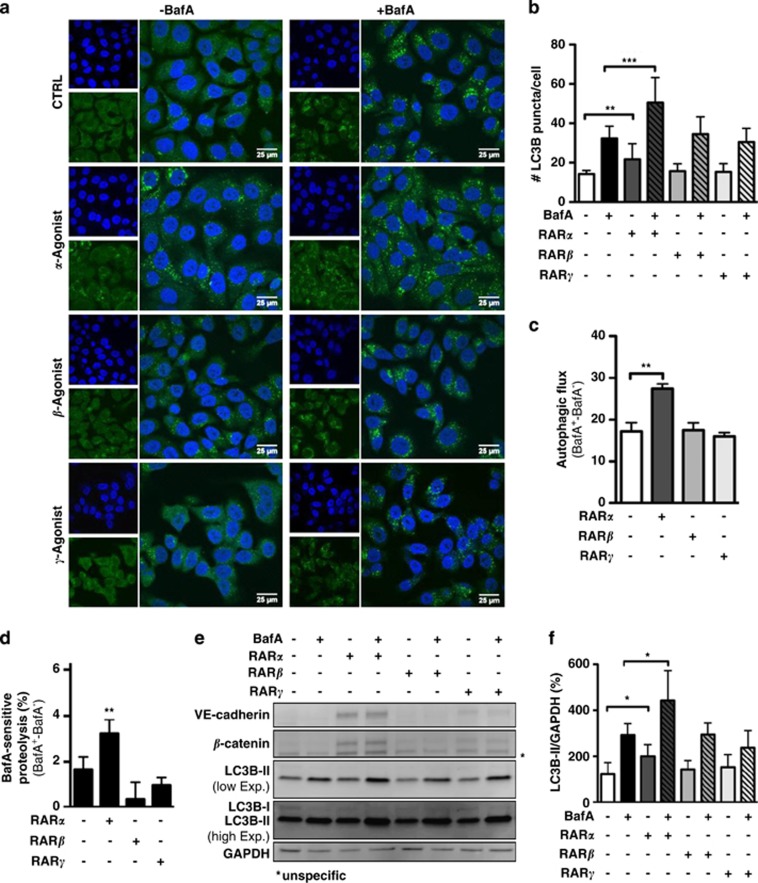
RAR*α* but not RAR*β* and RAR*γ* agonists induce autophagic flux in SKBR3 cells. (**a**) SKBR3 cells were treated with 1*μ*M of RAR*α*, RAR*β* or RAR*γ* agonists for 2 days in the presence and absence of BafA during the last 2 h, before subjection to immunofluorescence for LC3B. (**b**) Quantification of LC3B puncta from the experiment described in (**a**). Three independent experiments were quantified as described in Schläfli *et al.*^41^ (**c**) Autophagic flux was determined from the immunofluorescence analysis shown in (**a**). *N*=3, Student's *t*-test and **P<0.01. (**d**) Long-lived protein degradation assay of control or cells treated with 1 *μ*M of RAR*α*, RAR*β*, RAR*γ* agonists in the presence or absence of 200 nM BafA. Radioactivity was determined by liquid scintillation counting of four independent experiments. Data are shown as BafA-sensitive proteolysis. (**e**) Western blot analysis for VE-cadherin, *β*-catenin and LC3B of SKBR3 cells treated as in (**a**). (**f**) LC3B-II levels on western blot were normalized to GAPDH and quantified from at least five independent experiments using the ImageJ software (NIH, Bethesda, MD, USA). LC3B-II levels of control treated cells were arbitrarily set to 100%. Mann–Whitney *U-*test: *P<0.05, **P<0.01 and ****P<0.0001

**Figure 4:**
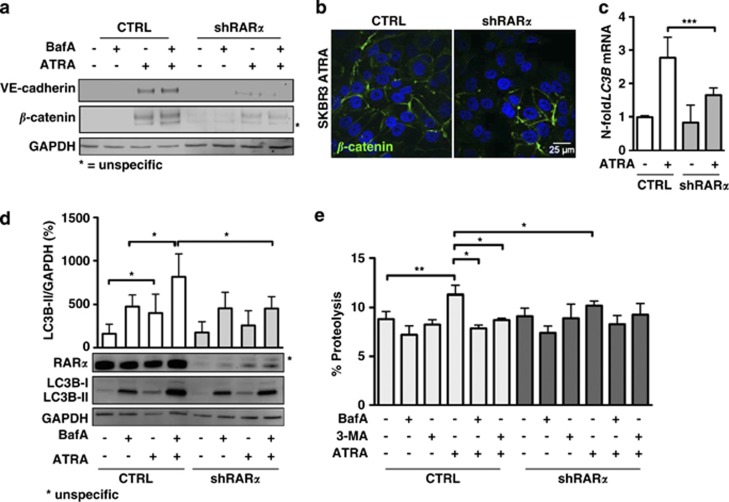
Depletion of RAR*α* prevents ATRA-induced autophagic flux in SKBR3 cells. (**a**) *β*-Catenin, VE-cadherin and GAPDH western blot analysis of SKBR3 cells transduced with a scrambled shRNA (CTRL) or RAR*α*-specific shRNA (shRAR*α*) treated with ATRA for 2 days in the presence and absence of 200 nM BafA during the last 2 h. (**b**) Immunohistochemistry of *β*-catenin in control and shRAR*α*-transduced cells subjected to 1 *μ*M ATRA for 4 days. Pictures were taken on a confocal microscope. (**c**) LC3B qPCR analysis of control and ATRA-treated SKBR3 cells expressing a scramble shRNA or shRAR*α*. Raw Ct values were normalized to ABL-1 mRNA and to the vehicle-treated control cells (^ΔΔ^Ct method). (**d**) LC3B western blotting and quantification of the autophagic activity in control and RAR*α*-knockdown SKBR3 cells upon treatment with 1 *μ*M ATRA for 2 days in the presence or absence of BafA for 2 h at 200 nM. LC3B-II expression was normalized to GAPDH and to vehicle-treated control cells. Standard deviations from at least five independent experiments are shown. (**e**) Long-lived protein degradation assay of control and ATRA-treated SKBR3 cells transduced with a scrambled shRNA (CTRL) or shRAR*α* in the presence or absence of BafA (200 nM) or 3-MA (10 mM) during the chase phase. Radioactivity was determined by liquid scintillation counting in three independent experiments. Mann–Whitney *U-*test: *P<0.05, **P<0.01 and ***P<0.001

As we observed increased autophagic flux only in ATRA-sensitive cells, we were interested in the signal-transduction mechanism leading to the observed phenotype. We focused on the main retinoic acid effectors, that are, the nuclear retinoid receptors, RAR*α*, RAR*β* and RAR*γ*. To this end, we treated SKBR3 cells with RAR*α*, RAR*β* and RAR*γ* agonists alone and in combination with BafA and determined LC3B puncta formation (Figures 3a and b). Quantification of the number of dots per cells revealed a significant increase of autophagic activity only when SKBR3 cells where subjected to the RAR*α* agonist AM580 (4-[[(5,6,7,8-tetrahydro-5,5,8,8-tetramethyl-2-naphthalenyl)carbonyl]amino]-benzoic acid; Figures 3b and c). To validate the LC3B immunofluorescence data, we additionally performed long-lived protein degradation assays. In agreement with the immunofluorescence data, only the specific RAR*α* agonist, AM580, significantly increased autophagic-dependent degradation of ^14^C-valine-labeled proteins (Figure 3d). As a third method to determine autophagic flux, LC3B-II western blot was performed (Figures 3e and f). Again, increased autophagic flux was seen only upon RAR*α* but not upon RAR*β* or RAR*γ* activation (Figures 3e and f). These results reinforce the notion that RAR*α* is the limiting nuclear receptor in the induction of autophagic flux by retinoic acid. It is worth mentioning that autophagy induction by AM580 was paralleled by induction of the differentiation markers *β*-catenin and VE-cadherin (Figure 3e). To support our hypothesis that autophagy is mediated by RAR*α*, we depleted RAR*α* in SKBR3 cells. As observed by Bosh *et al.*,^13^ knocking down RAR*α* changed the cell morphology to a spindle-like shape (data not shown). Depletion of RAR*α* prevented the ATRA-dependent increase in *β*-catenin and VE-cadherin, confirming that RAR*α* activation is key to cytodifferentiation (Figure 4a).^20^ In addition, ATRA-dependent cell membrane relocalization of *β*-catenin observed in control transduced cells was reduced in SKBR3 RAR*α* knockdown cells (Figure 4b). Although RAR*α* depletion did not affect the basal autophagy levels, it significantly blocked LC3B mRNA induction as well as LC3B-II turnover following ATRA treatment (Figures 4c and d). Finally, RAR*α* depletion significantly decreased BafA- and 3-MA-dependent degradation of long-lived proteins in ATRA-treated cells (Figure 4e). Taken together, these results strongly suggest that ATRA-induced autophagy is mediated by RAR*α*.

### ATG gene depletion or chloroquine-mediated autophagy inhibition enhances apoptosis in ATRA-treated SKBR3 cells

**Figure 5:**
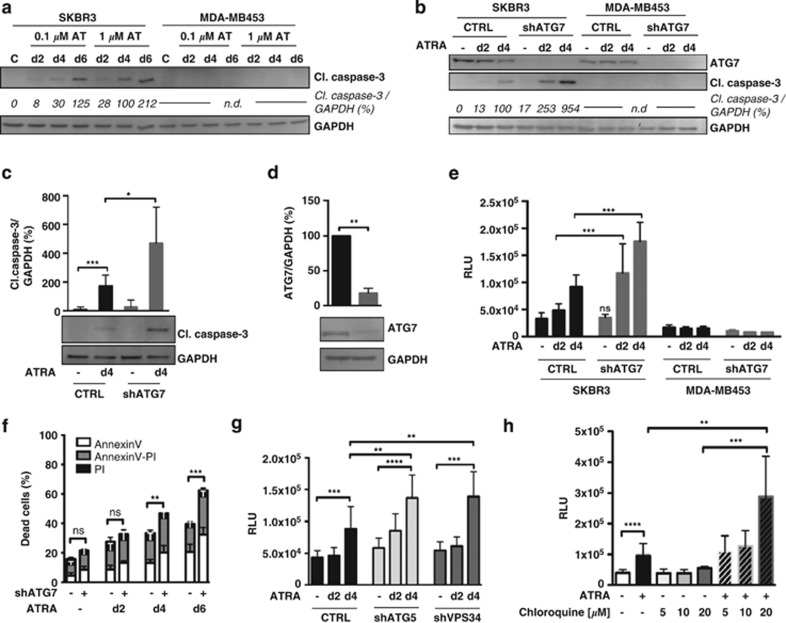
ATG gene depletion and pharmacological inhibition of autophagy significantly enhances apoptosis in ATRA-treated SKBR3 cells. (**a**) SKBR3 and MDA-MB453 cells were treated with increasing concentrations of ATRA (AT) for 2, 4 and 6 days as indicated. Western blot analysis for cleaved (cl.) caspase-3 and GAPDH was performed. ImageJ software was used to quantify bands. Raw values were normalized to GAPDH. Caspase-3 levels of SKBR3 cells treated with 1 *μ*M ATRA for 4 days were arbitrarily set to 100% as expression levels at day 0 or 2 were undetectable or weak, respectively. (**b**) ATG7 and cleaved caspase-3 western blot analysis of control or shATG7-transduced SKBR3 and MDA-MB453 cells treated with 1 *μ*M ATRA for 2 and 4 days. Cleaved (cl.) caspase-3 expression levels were quantified as in (**a**). (**c**) Western blot analysis and quantification of cleaved (cl.) caspase-3 in control and shATG7-transduced SKBR3 cells treated with 1 *μ*M ATRA for 4 days. Raw values for cleaved capsase-3 were normalized to GAPDH using the ImageJ software. Vehicle-treated control cells were arbitrarily set to 100%. Bars and s.d. are representative of five independent experiments. (**d**) Knockdown efficiency of ATG7 and statistics thereof. Normalization was performed as described in (**c**). (**e**) Caspase-3/7 activity was measured using Caspase-Glo-3/7 assay from Promega. RLU values were calculated according to manufacture's recommendations and normalized to the cell number. Values are shown for SKBR3 and MDA-MB453 control or shATG7-transduced cells treated with 1 *μ*M ATRA for 2 and 4 days, respectively. Standard deviations are shown from three independent experiments. (**f**) Annexin V/PI fluorescence-activated cell sorting (FACS) analysis of control or ATG7-depleted SKBR3 cells at day 2, 4 and 6 of treatment with 1 *μ*M ATRA. Bars represent five independent experiments. (**g**) Control, ATG5- and VPS34 SKBR3- knockdown cells were treated and analyzed as in (**e**). (**h**) Caspase-3/7 activity of SKBR3 cells treated with ATRA, increasing concentrations of chloroquine or a combination thereof as indicated. Analysis as in (**e**). Mann–Whitney *U-*test: *P<0.05, **P<0.01, ***P<0.001 and ****P<0.0001

The antitumor effects of ATRA on SKBR3 cells are mainly the result of a growth inhibitory and cytodifferentiating response with subsequent induction of apoptosis.^20^ Indeed, we found a time- and dose-dependent increase in caspase-3 cleavage in SKBR3 but not in MDA-MB-453 cells treated with ATRA (Figure 5a and Supplementary Figure 2a). To evaluate whether depletion of key autophagy genes affects ATRA-induced cell death, we first silenced ATG7 in SKBR3 and MDA-MB453 cells. Control and ATG7-depleted cells were subjected to ATRA treatment before apoptosis was determined at different time points using multiple assays. In SKBR3 cells, knocking down ATG7 clearly enhanced ATRA-dependent cleavage of caspase-3. This phenomenon was particularly pronounced at day 4 (Figures 5b and c). In contrast, no induction of cleaved caspase-3 was evident in either control or ATG7-silenced MDA-MB453 cells (Figure 4b, right panel). ATG7-knockdown efficiency was evaluated by western blotting (Figure 5d). In agreement with the western blot results, caspase-3/7 activity was significantly enhanced in ATRA-treated SKBR3 ATG7-knockdown cells, whereas silencing ATG7 in MDA-MB453 cells did not affect this enzymatic activity (Figure 5e). Increased Annexin V/PI staining in ATG7-depleted SKBR3 cells as compared with control cells validated our previous observations (Figure 5f). The high sensitivity of the Annexin V assay allowed the detection of increased basal levels of apoptosis even in untreated SKBR3 ATG7-depleted cells, although not statistically significant. Based on our findings above that RAR*α* is key in activating the autophagic flux, we repeated our experiments in ATG7-knockdown and control SKBR3 cells using the RAR*α* agonist AM580. As observed with ATRA, silencing ATG7 significantly enhanced AM580-induced SKBR3 cell death (Supplementary Figures 2b and c). To support our findings with SKBR3 ATG7-knockdown cells above, we silenced two additional ATG genes, *ATG5* and the class III phosphatidylinositol 3-kinase *VPS34* in these cells. Following ATRA treatment, *ATG5* and *VPS34* depletion led to a consistent and significant increase in SKBR3 caspase-3/7 activity as compared with scramble control transfected cells (Figure 5g). Knockdown efficiencies for ATG5 and *VPS34* are shown in Supplementary Figure 2d. This increased cell death response was paralleled by attenuated activation of autophagy as assessed by *WIPI1* transcript levels (Supplementary Figure 2e).^23^ Finally, we evaluated the action of chloroquine, a clinically relevant autophagy inhibitor, on SKBR3 cells exposed to ATRA. We observed no marked increase in cell death when SKBR3 cells were treated with chloroquine alone, but in combination with ATRA, a significant increase in cell death compared with that in cells treated with ATRA alone was seen (Figure 5h).

**Figure 6:**
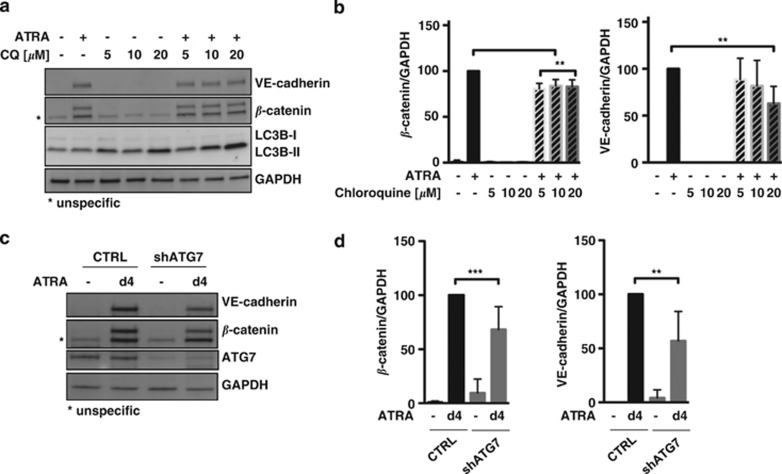
Inhibition of autophagy impacts on ATRA-induced epithelial differentiation of SKBR3 cells. (**a**) SKBR3 cells were treated for 4 days with 1 *μ*M ATRA, increasing concentrations of chloroquine or a combination thereof as indicated before western blot analysis for VE-cadherin, *β*-catenin, LC3B and GAPDH. (**b**) Quantification of *β*-catenin and VE-cadherin western blots as one is shown in (**a**). Raw values were normalized to GAPDH and levels of ATRA-treated control transduced cells were arbitrary set to 100% as signals were not always detectable in vehicle-treated control cells. Bars and s.d. representative of five independent experiments. (**c**) SKBR3 control and ATG7-depleted cells were subjected to 1 *μ*M ATRA for 4 days and expression levels of VE-cadherin, *β*-catenin, ATG7 and GAPDH were determined by western blot. (**d**) Quantification of western blots for *β*-catenin and VE-cadherin. Normalization was performed as described in (**b**)

As induction of autophagy was paralleled by epithelial differentiation (Figures 1a and 3d), we were asking whether inhibition of autophagy would impact on differentiation. To this end, we treated SKBR3 cells with ATRA, chloroquine or a combination thereof for 4 days and determined the expression of the differentiation markers VE-cadherin and *β*-catenin by western blotting. Blocking autophagy markedly attenuated epithelial differentiation (Figure 6a–d). Whereas the effects of autophagy inhibition on *β*-catenin levels, although significant, were rather modest (Figure 6b, left panel), the impact on VE-cadherin were more pronounced at least when cells were treated with ATRA and 20 *μ*M chloroquine (Figure 6b, right panel). Similarly, depletion of ATG7 significantly decreased ATRA-induced epithelial differentiation of SKBR3 cells (Figures 6c and d). Interestingly, steady-state levels of *β*-catenin and VE-cadherin were increased in ATG7-depleted cells, although the effect is not statistically significant.

In conclusion, our results demonstrate that inhibition of autophagy either by pharmacological or genetic means significantly increases ATRA-mediated cell death in retinoid-sensitive SKBR3 cells and lowers epithelial differentiation.

## Discussion

In the present study, we report that ATRA induces autophagic flux in retinoid-sensitive SKBR3 breast cancer cells via RAR*α*. In contrast, autophagy activation is not observed in the retinoid-resistant MDA-MB453 cell line. Using 3-MA as a specific inhibitor of macroautophagy,^21^ we were able for the first time to directly link macroautophagy induction to RAR*α* activation in breast cancer cell lines. We hypothesize that ATRA induces autophagy only in breast cancer cells that are not terminally differentiated and can be differentiated by retinoids for the following reasons: (1) selective activation of RAR*α* by AM580 or ATRA is sufficient to induce both autophagy and cytodifferentiation^20^ in SKBR3 cells, and (2) MDA-MB453 cells are resistant to ATRA-dependent autophagy and differentiation. In agreement with our hypothesis, ATRA causes autophagy in differentiating AML cells.^9, 10, 11, 12^ Additionally, NB4-resistant cells (NB4-R2), which are unable to differentiate upon ATRA administration, do not show an autophagic response to retinoids.^11^ A study by Anguiano *et al.*^24^ demonstrated that ATRA does not promote macroautophagy in mouse fibroblasts, although RAR*α* knockdown decreased macroautophagy under basal conditions in these terminally differentiated cells. In contrast to their study, where depleting RAR*α* induced 3-MA-sensitive autophagy, our results in differentiation-competent breast cancer cells show that RAR*α* depletion abrogates 3-MA-sensitive proteolysis. Work performed in HeLa cells, a cell type that cannot be further differentiated with ATRA, indicated that retinoic acids promote autophagosome maturation through a pathway that does not involve the classic nuclear retinoid receptor pathway.^25^ This further supports our hypothesis that RARs are particularly important for retinoid-mediated autophagy induction in non-terminally differentiated cells.

A novel treatment strategy for breast cancer may involve combinations of retinoids and autophagy inhibitors with the aim of shortening the survival of breast cancer cells. Several groups identified a role for autophagy in response to anticancer therapy, for example, camptothecin activates autophagy, which in turn delays apoptotic cell death in noninvasive breast cancer.^26^ Somewhat conflicting data exist on the role of autophagy activation during HER2-targeted therapy with lapatinib. On the one hand, combining lapatinib with a pharmacological inhibitor of autophagy resulted in reduced cell death,^27^ whereas on the other blocking autophagy by beclin-1 depletion potentiated the therapeutic efficiency of lapatinib.^28^ Furthermore, inhibition of epirubicin-triggered autophagy enhances therapeutic efficiency in MDA-MB-231 breast cancer cells.^29^ Given the possible role for autophagy as therapeutic strategy, it is crucial to emphasize that different stress inducers lead either to a cytoprotective or cytotoxic autophagy response. The outcome is clearly dependent on the stage and type of tumor.^30^ These findings underline the importance to evaluate the tumor response to autophagy individually for each type of cancer and therapy condition. Our results clearly support a model where blocking autophagy by genetic and pharmacological means greatly improves ATRA toxicity in SKBR3 cells. Therefore, it is tempting to speculate that ATRA-induced autophagy is cytoprotective in our cell line model. However, careful interpretation of these results is warranted, as one of our cell death assays showed enhanced basal levels of apoptosis in control treated SKBR3 cells. We cannot finally exclude that SKBR3 cells are highly sensitive to autophagy inhibition irrespective of ATRA treatment. Another explanation might be that physiological concentrations of ATRA, present in the fetal bovine serum (FBS), are sufficient to induce some degree of cell death upon autophagy inhibition. The exact mechanism how blocking autophagy contributes to cell death in ATRA-sensitive breast cancer cells remains to be elucidated.

Autophagy and cellular differentiation have been linked earlier, for example, during the last step of reticulocyte maturation, all membrane-bound organelles and ribosomes are removed to ensure optimal hemoglobin production and oxygen transport.^31^ Autophagy has been shown to contribute to the elimination of mitochondria in this step.^32, 33, 34^ Similarly, adipocytes induce autophagy, which is important for the remodeling process.^3^ Furthermore, ATG5 is crucial for the transition from the pro- to the pre-B-cell stage,^35^ and monocyte differentiation is accompanied by autophagy induction.^36, 37^ Strategies aiming at the reactivation of differentiation programs in the stem cell compartment of solid tumors have great therapeutic potential.^38^ For example, CD44 inhibition in breast cancer stem cells results in differentiation and enhanced drug sensitivity.^39^ Similarly, ATRA-dependent differentiation of cancer stem cells reduces the formation of mammospheres and the tumor-initiating potential of certain breast cancer cell lines.^40^ We now show that autophagy once activated supports epithelial differentiation, as assessed by *β*-catenin and VE-cadherin expression. These markers are significantly downregulated upon inhibition of autophagy. In this regard, autophagy inhibition as suggested above seems to be rather disadvantageous, as it decreases the effects of ATRA on differentiation. Nevertheless, the negative effect of blocking autophagy on differentiation might be negligible given the benefits of highly increased cell death when these two treatments are combined.

## Materials and Methods

### Chemicals

AM580 is an RAR*α* agonist, BMS641 (3-chloro-4-[(E)-2-(5,5-dimethyl-8-phenyl-6H-naphthalen-2-yl)ethenyl]benzoic acid; also known as UV2003) is a RAR*β* agonist, whereas CD437 (6-(4-hydroxy-3-tricyclo[3.3.1.1^3,7^]dec-1-ylphenyl)-2-naphthalenecarboxylic acid) is an RAR*γ* agonist. AM580 and CD437 were purchased from Tocris Inc. (Bristol, UK), whereas BMS641 was a kind gift of Dr. Angel R deLera (Departamento de Química Orgánica, Universidade de Vigo, Pontevedra, Spain). 3-MA (no. S2767)/BafA (B1793) and chloroquine (no. C6628) were purchased from Sigma-Aldrich (Buchs, Switzerland) and Seleckchem (Huston, TX, USA), respectively.

### Cell lines and culture conditions

Human breast cancer lines MDA-MB-453 and SKBR3 were cultured in DMEM/F12 supplemented with 5% FBS, 50 U/ml penicillin and 50 *μ*g/ml streptomycin in humidified atmosphere containing 5% CO_2_ at 37 °C. ATRA- and the RAR-specific agonists were used at 0.1 or 1 *μ*M.

### Long-lived protein degradation assay

Cells were seeded in 24-well plates and radiolabeled with 0.2 *μ*Ci ^14^C-valine (l-[U-14-C]valine; code CFB.75; Amersham, Glattbrugg, Switzerland) per ml per well for 2 days in combination with ATRA or agonist/antagonists. Cells were washed with PBS to remove free radioactivity, and chased in 0.5 ml complete DMEM/F12 supplemented with 10 mM cold valine for 1 h. Short-lived proteins were washed out and cells were treated for additional 5.5 h in complete control medium DMEM/F12 supplemented with 200 nM BafA or DMSO as control. Cells were scraped in their media and further processed as described in Brigger *et al.*^11^ with the following changes: after the cold chase phase, DMSO- and ATRA-treated cells were incubated in complete control medium DMEM/F12 in the presence or absence of 200 nM BafA or 10 mM 3-MA. Radioactivity was determined by liquid scintillation counting in triplicates. The degradation rate for long-lived proteins was calculated as the percentage of radioactivity in the TCA-soluble fraction relative to the total radioactivity in non-soluble fractions. To calculate the BafA-sensitive autophagy, the values were subtracted from the corresponding sample treated with BafA.

### Western blotting

Whole-cell extracts were prepared using 8 M urea buffer with 0.5% Triton X-100, supplemented with proteinase inhibitor cocktail (complete; Roche, Basel, Switzerland). Total protein (25–80 *μ*g) was separated on a 12% SDS-polyacrylamide gel and transferred to a PVDF membrane. Blots were incubated with primary antibody overnight at 4 °C, washed and incubated with secondary anti-mouse or anti-rabbit antibodies for 1 h at room temperature protected from light. Primary antibodies used were anti-LC3B (NB600-1384; Novus Biologicals, Abingdon, UK), cleaved caspase-3 (9661; Cell Signaling, Danvers, MA, USA), VE-CAD (no. 2158; Cell Signaling), *β*-catenin (9562; Cell Signaling), VPS34 (no. NB110-87320SS; Novus Biologicals), RAR*α* (c-551; Santa Cruz, Heidelberg, Germany) and anti-glyceraldehyde 3-phosphate dehydrogenase (GAPDH) (MAB374; Milipore, Vienna, Austria). Secondary antibodies were used as described in Brigger *et al.*^11^

Generation of knockdown and Cherry-GFP-LC3B-expressing cell lines pLKO.1 lentiviral vectors expressing small hairpin RNAs (shRNAs) targeting RAR*α*, ATG7, ATG5 and VPS34 or a non-targeting shRNA control (SHC002) were purchased from Sigma-Aldrich. A lentiviral vector expressing Cherry-GFP-LC3B was provided by Dr. MS Soengas (Melanoma Laboratory, Madrid, Spain). Lentiviral production and transduction of SKBR3 cells was carried out as described.^11^ Transduced cell populations were selected for 4 days using 1.5 *μ*g/ml puromycin.

### Immunofluorescence

Cells were fixed with 4% paraformaldehyde (PFA) or with ice-cold 100% methanol for 4 min and then washed with PBS containing 0.5% glycine. Subsequently, cells were permeabilized with Triton X-100 for PFA fixed cells. Anti-*β*-catenin (9562; Cell Signaling) and anti-LC3B (3868; Cell Signaling) antibody were incubated for 1 h at room temperature followed by washing steps with PBS containing 0.1% Tween (PBS-T). Cells were incubated with the secondary antibody (anti-rabbit, 111-096-045; Jackson ImmunoResearch, West Grove, PA, USA) for 1 h at room temperature. Prior mounting in fluorescence mounting medium (S3032; Dako) cells were washed three times with PBS-T. Fluorescence microscopy of GFP-Cherry-LC3B expressing SKBR3 and MDA-MB-453 human breast cancer cell lines was performed as described in Brigger *et al.*^11^ Images were taken on a confocal microscope Olympus FluoView-1000 (Olympus, Volketswil, Switzerland) at x63 magnification.

### Annexin V/PI FACS analysis

Cells where harvested at indicated time points, washed using Annexin V buffer (BD Biosciences, Allschwil, Switzerland) and stained with Annexin V-FITC (BioLegend, Fell, Germany). Prior FACS analysis, PI (Sigma-Aldrich) was added at a final concentration of 2.5 *μ*g/ml. Analysis was acquired on an LSR II (BD Biosciences) and analyzed using the FlowJo software.

### Caspase-3/7 activity assay

Caspase-3/-7 activity was determined using the Caspase-Glo-3/7 assay (Promega, Dübendorf, Switzerland) according to the manufacturer's description. As cell number greatly differed between treated and untreated cells, relative luminescence values (RLUs) were normalized to results obtained from an Alamarblue assay (LifeTechnologies, Zug, Switzerland).

### Real-time qRT-PCR

RNA extraction, RT-PCR and real-time qRT-PCR (qPCR) and data analysis were performed as described.^11^ Gene Expression Assay for *WIPI1* and *MAP1LC3B* (microtubule-associated protein 1 light chain 3) used in a 96-well format on the StepOne plus sequence detection system were Hs00215872_m1 and Hs00797944_s1, respectively (Applied Biosystems, Rotkreuz, Switzerland). *HMBS* *ABL1* primers where 5′-TGGAGATAACACTCTAAGCATAACTAAAGGT-3′ and 5′-GATGTAGTTGCTTGGGACCCA-3′ and probe was 5′-FAM-CCATTTTTGGTTTGGGCTTCACACCATT-TAMRA-3′.

## Abbreviations

ATRA: all-*trans* retinoic acid
RAR: retinoic acid receptor
APL: acute promyelocytic leukemia
HER2: human epidermal growth factor receptor-2
LC3 (MAP1LC3): microtubule-associated protein 1 light chain 3
BafA1: bafilomycin A1
GAPDH: glyceraldehyde 3-phosphate dehydrogenase
3-MA: 3-methyladenine
shRNA: small hairpin RNA
ATG: autophagy-related
