# Supplementary figures and images for: Activation of RARα induces autophagy in SKBR3 breast cancer cells and depletion of key autophagy genes enhances ATRA toxicity
Source: Cell Death Dis. 2015 Aug 27;6(8):e1861–. doi: 10.1038/cddis.2015.236 (PMC4558517; doi:10.1038/cddis.2015.236)

Supplementary figure 1

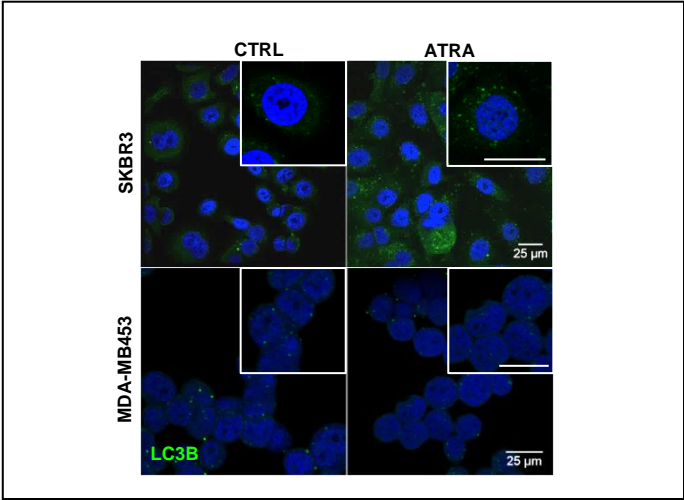

Supplement: Supplementary Figure 1 [file cddis2015236x1.pdf]

Supplementary figure 2

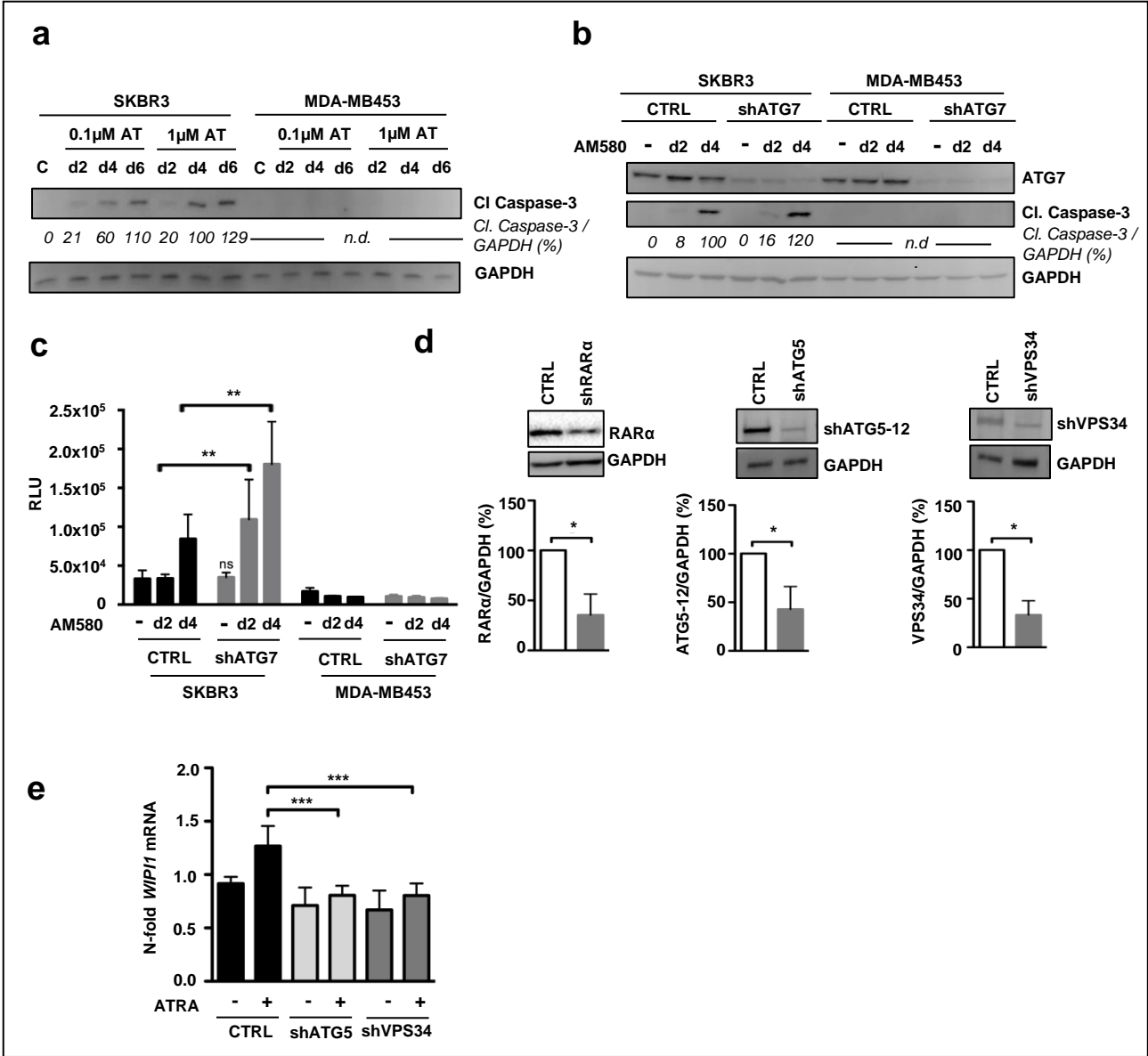

Supplement: Supplementary Figure 2 [file cddis2015236x2.pdf]
