# Supplementary material for: Activation of RARα induces autophagy in SKBR3 breast cancer cells and depletion of key autophagy genes enhances ATRA toxicity
Source: Cell Death Dis. 2015 Aug 27;6(8):e1861–. doi: 10.1038/cddis.2015.236 (PMC4558517; doi:10.1038/cddis.2015.236)
Supplement: Supplementary Figure Legends [file cddis2015236x3.doc]

**Supplementary Figure 1. Increased LC3B puncta during ATRA therapy**. (**a**) Staining of endogenous LC3B in SKBR3 and MDA-MB-453 cells treated with 1μM ATRA for 2 days.

**Supplementary Figure 2.** **ATG7 depleted SKBR3 cells show increased sensitivity to ATRA and the RARα agonist AM580**. **(a)** SKBR3 and MDA-MB-453 were treated as in Figure 5a. Western blot analysis for cleaved Caspase-3 was performed. GAPDH was used as a loading control. Cleaved Caspase-3 levels of SKBR3 control transduced cells treated with 1μM ATRA for 4 days were arbitrarily set to 100%. **(b)** SKBR3 and MDA-MB-453 ATG7 knockdown and the respective control cells were treated with 1μM RARα agonist AM580 for 2 and 4 days. Western blot analysis of ATG7, cleaved capsase-3 and GAPDH protein levels is shown. Analysis as in a. **(c)** SKBR3 and MDA-MB-453 ATG7 knockdown and the respective control cells were treated as in c. Caspase-3/7 activity was determined using Caspase-Glo® 3/7 assay data are given as relative luminescence values (RLU). Mann-Whitney-U test: **P<0.01.

**(d)** RARα , ATG5 and VPS34 Western blot analysis of control and the respective SKBR3 knockdown cells. GAPDH was used as a loading control. **(e)** WIPI-1 qPCR analysis of control, ATG5 and VPS34 knockdown SKBR3 cells upon ATRA treatment for 2 days. Normalization as in 4c.
